# Investigating the Role of CTCs with Stem/EMT-like Features in Metastatic Breast Cancer Patients Treated with Eribulin Mesylate

**DOI:** 10.3390/cancers14163903

**Published:** 2022-08-12

**Authors:** Maria A. Papadaki, Anastasia Mala, Aikaterini C. Merodoulaki, Maria Vassilakopoulou, Dimitrios Mavroudis, Sofia Agelaki

**Affiliations:** 1 Laboratory of Translational Oncology, School of Medicine, University of Crete, 71003 Heraklion, Greece; 2 Department of Medical Oncology, University General Hospital of Heraklion, 71500 Heraklion, Greece

**Keywords:** eribulin mesylate, circulating tumor cells (CTCs), epithelial-to-mesenchymal transition (EMT), cancer stem cells (CSCs), liquid biopsy, breast cancer, CTC monitoring, breast cancer (BC), biomarkers, resistance

## Abstract

**Simple Summary:**

Eribulin mesylate, an anti-mitotic drug used for the treatment of metastatic breast cancer (BC), exhibits significant effects on cancer cell migration, invasion, and metastatic seeding in experimental models. Interestingly, eribulin treatment has been shown to target the cancer stem cell (CSC) subsets in vitro and reverse the epithelial-to-mesenchymal transition (EMT) state of BC cells. In the current study, circulating tumor cells (CTCs) identified in the peripheral blood of patients with metastatic BC were analyzed at different time points during eribulin treatment and on disease progression. The results contribute new data on the mechanisms of resistance to eribulin mesylate and the prognostic relevance of CTC analyses for eribulin-treated metastatic BC.

**Abstract:**

We herein aimed to assess the effect of eribulin mesylate on the cancer stem cell (CSC)/EMT-like phenotype of CTCs, and to investigate the prognostic role of CTC detection and monitoring for eribulin-treated BC patients. Peripheral blood was obtained at baseline (*n* = 42 patients) and 8 days after treatment initiation (C1D8: *n* = 22), and on disease progression (PD: *n* = 26). PBMCs cytospins were immunofluorescently stained for Cytokeratins/ALDH1/TWIST1/DAPI and analyzed via Ariol microscopy. CTCs were detected in 33.3%, 27.3%, and 23.1% of patients at baseline, C1D8, and PD, respectively. Accordingly, partial-EMT+ CTCs represented 61.3%, 0%, and 37.5% of total CTCs, whereas the CSC-like phenotype was consistently expressed by 87.5%, 75%, and 91.7% of CTCs at the respective time points. Interestingly, the CSC+/partial-EMT+ subset prevailed at baseline, but it was eradicated on C1D8 and resurged again during PD. CTC detection at baseline was associated with reduced PFS (*p* = 0.007) and OS (*p* = 0.005), and was an independent risk factor for death (HR: 3.779, *p* = 0.001; multivariate analysis). The CSC+/partial-EMT+ CTCs emerged as the only subset with adverse prognostic significance, while CTC monitoring during eribulin therapy improved the prediction of disease progression. These results indicate that resistant CTC subsets persevere eribulin treatment and highlight the prognostic implications of CTC analyses for eribulin-treated BC patients.

## 1. Introduction

Breast cancer (BC) accounts for one in six cancer-related deaths among women worldwide [1]. Metastasis remains the leading cause of death for BC patients; hence the understanding of the biology underlying the metastatic process is crucial for the development of therapeutic strategies to improve the management of BC [2]. During metastasis, tumor cells are detached from the primary tumor, enter the bloodstream, migrate to distant sites, and finally extravasate to a new organ to develop a secondary tumor [3]. The detection of circulating tumor cells (CTCs) in the peripheral blood (PB) of patients with BC has emerged as a promising prognostic tool associated with a high risk for disease progression and death [4]. Moreover, the longitudinal CTC analysis over the course of treatment can provide additional information on the disease status in real-time [5]. Recent data also support the role of CTC assessment in guiding first-line therapy for metastatic BC [6]. Nevertheless, beyond CTC detection, the genotypic and phenotypic analysis of these cells is essential to uncover mechanisms and pathways driving metastasis and resistance to current therapies [3,7].

Accumulating evidence supports that tumor cells bearing cancer stem cell (CSC) and partial-epithelial-to mesenchymal transition (EMT) properties display a survival advantage, increased invasion, and migration capacities, and drug resistance [8]. During the last decade, we, among others, revealed that CTCs of BC patients frequently exhibit CSC-like and partial-EMT features [9,10,11,12]. Our studies suggest that CTCs expressing putative CSC+/partial-EMT+ phenotypes prevail in patients with metastatic as compared to early-stage BC [11,13] and that their incidence is further increased after the administration of first-line chemotherapy [14]. Importantly, the detection of CSC+/partial-EMT+ CTCs emerged as a prognostic factor associated with an unfavorable outcome for patients with metastatic BC, especially for those with HER2-negative disease [14]. These findings collectively suggest that CSC+/partial-EMT+ CTCs constitute an aggressive cell population that exhibits resistance to current chemotherapy drugs.

Eribulin mesylate is a non-taxane microtubule-targeting agent that significantly inhibits cancer cell migration, invasion, and metastatic seeding in experimental models [15]. Preclinical evidence derived from BC cell lines also suggests that eribulin exhibits anti-CSC activity [16] and induces the reversal of EMT on tumor cells [15]. These certain mechanisms of action may provide an explanation for the demonstrated efficacy of eribulin mesylate in chemotherapy-resistant metastatic BC. Specifically, results from phase III randomized trials support that eribulin treatment significantly improves the overall survival (OS) of heavily pre-treated patients with metastatic BC [17,18]. Pooled analysis of these studies further revealed a significant increase in OS with eribulin for patients who had received at least two prior lines of chemotherapy, including anthracyclines and taxanes, and especially those with HER2-negative and triple-negative BC [19]. Recent findings of a randomized phase II study also support that eribulin as first-line or second-line chemotherapy provided an important clinical benefit to patients with recurrent HER2-negative BC [20]. However, effective and readily available predictive biomarkers for eribulin treatment are still lacking, while the mechanisms involved in eribulin resistance were only recently investigated and require further elucidation [21,22].

We herein hypothesized that eribulin mesylate could have an impact on the stemness and EMT state of CTCs from BC patients and that CTC analyses may have a role as a prognostic tool for patients treated with eribulin. To explore these hypotheses, we performed a longitudinal analysis of CTCs and their CSC/EMT-like phenotypes from BC patients prior to the start and eight days after the administration of the first cycle of eribulin treatment, as well as on disease progression. Our results provide for the first time evidence for the effect of eribulin treatment on the CSC/EMT features of CTCs and point towards potential resistant CTC subsets. Importantly, the current study reveals the prognostic relevance of CTC detection and monitoring in patients with metastatic BC treated with eribulin mesylate.

## 2. Materials and Methods

### 2.1. Patients and Treatment

Forty-two patients with metastatic BC, who received treatment with eribulin mesylate at the Department of Medical Oncology, University General Hospital of Heraklion, Greece, between 2016 and 2020 were included in the study. All patients received eribulin treatment for stage IV metastatic disease.

Eribulin was administered intravenously, over 2 to 5 min, at the recommended dose of 1.23 mg/m^2^ on days 1 and 8 of a 21-day cycle, until disease progression. The full drug dosage was administered to 34 out of 42 patients. Three patients received a reduced dose of 0.97 mg/m^2^ at the start of treatment, whereas 3 and 2 patients had a dose reduction at the second and fourth cycle, respectively, based on the dosage adjustment for hematological and non-hematological toxicity provided in the summary of product characteristics (SPC) of the drug.

For CTC analyses, peripheral blood (PB) samples were obtained before the initiation of eribulin treatment (baseline) (no of patients: *n* = 42). Paired PB samples were additionally collected on day 8 of the first cycle of treatment (C1D8) (no of patients: *n* = 22) and on disease progression (PD) (no of patients: *n* = 26). Clinical characteristics and follow-up information were prospectively collected. Consecutive patients with available blood samples were selected based on the following criteria: age over 18 years old, histologically diagnosed BC, absence of secondary malignancies, available written informed consent, and complete clinical and pathological data.

### 2.2. Enrichment of CTCs

PB samples (10 mL in EDTA) were enriched for CTCs through the isolation of peripheral blood mononuclear cells (PBMCs) via Ficoll–Hypaque density gradient (d = 1.077 g/mL; Sigma-Aldrich, St. Louis, MO, USA) centrifugation at 650 g for 30 min, as described in our previous reports [23]. Cytospins of 500,000 cells were prepared and stored at −80 °C until use.

### 2.3. Immunofluorescence (IF)

PBMC cytospins were immunofluorescently stained for Cytokeratins (CKs)/ALDH1/TWIST1 [11,12,14]. Briefly, the fixation of cells with PBS/Formaldehyde 3.7% and permeabilization with PBS/Triton X-100 0.1%) were followed by a 1 h blocking with PBS/1% BSA. Primary antibodies included Alexa Fluor 488-conjugated anti-CKs antibodies (clones: AE1/AE3, Thermo Fisher Scientific, Waltham, MA, USA and C11, Novus Biologicals) for CTC detection, anti-ALDH1 (Abcam, Cambridge, MA, USA) as a marker for CSC phenotype, and anti-TWIST1 (Abcam) as a marker for EMT phenotype. Secondary antibodies included Alexa Fluor 555- and Alexa Fluor 633-conjugated antibodies (Molecular Probes, Invitrogen), and DAPI antifade reagent (Molecular Probes, Invitrogen) was used for staining cell nuclei.

Cytospins of HepG2 cancer cells were included in all the IF stainings applied to patient samples and served as controls for the expression of CKs, ALDH1, and TWIST, as previously described [11,12,14].

### 2.4. Detection and Phenotypic Analysis of CTCs

A total of 2 × 10^6^ PBMCs (two slides) per patient were analyzed using the automated image analysis Ariol microscopy system and the Ariol system CTCs software (Genetix, Cambridge, UK) [23,24,25]. As described in our previous studies, CK expression was used to define CTCs, whereas high ALDH1 expression and nuclear TWIST1 localization were used to define the CSC+ and partial-EMT phenotypes, respectively, on CK+ CTCs. The low/absent ALDH1 expression was defined as a non-CSC phenotype, while the cytoplasmic/absent TWIST1 expression was characterized as an epithelial-like phenotype of CK+ CTCs [11,12,14]. The sensitivity and specificity of the methodology used for CTC characterization according to ALDH1 and TWIST1 have been previously demonstrated [11].

The detection of at least one CTC was used to define patient positivity for CTCs; in accordance, the detection of at least one CTC of a particular phenotype was used to define patient positivity for this phenotype [12,23,24,25].

### 2.5. Statistical Analysis

Statistical analyses were performed using IBM SPSS Statistics version 20. Fisher’s exact test was used to investigate possible correlations between the detection and phenotype of CTCs, and patient and disease characteristics. Wilcoxon *t*-test was used to compare CTC counts among different time points. Kaplan–Meier survival analysis was used to estimate the probability of relapse and death over time, and the log-rank test was used to compare the survival measures between groups. Progression-free survival (PFS) was calculated from the initiation of eribulin treatment until disease progression or death from any cause, whereas overall survival (OS) was calculated from the start of eribulin treatment to death from any cause. The median follow-up time was estimated by reverse Kaplan–Meier analysis. The Cox regression analysis was used for the prediction of PFS and OS; the variables with statistical significance in univariate analysis were included in a multivariate regression analysis, following the “one in ten” rule [24]. *P*-values were considered significant at the *p* < 0.05 level.

## 3. Results

### 3.1. Patient and Disease Characteristics

Patient and disease characteristics are summarized in Table 1. Response to treatment was non-evaluable in 7 patients who received only one or two cycles of treatment due to clinical deterioration. At the time of analysis, with a median follow-up of 31.6 months, 95%CI: 11.5–51.7), all 42 patients had progressed (median PFS: 2.5 months, 95%CI: 2.0–3.1) and 35 patients had died (median OS: 9.0 months, 95%CI: 5.6–12.4).

### 3.2. Detection and Monitoring of CTCs during Eribulin Treatment and on Disease Progression

CTCs (CK-positive cells) were detected in 14/42 patients (33.3%) at baseline before the start of treatment, in 6/22 patients (27.3%) on day 8 (C1D8), and in 6/26 patients (23.1%) on PD (Table 2). Among 19/42 patients (45.2%), CTCs were detected at any of the three time points. The total and mean numbers of CTCs identified at different time points are shown in Table 2.

Regarding the kinetics of CTCs between baseline and C1D8, their numbers increased, decreased, or remained unchanged in 5, 7, and 10 out of 22 patients, respectively. Moreover, CTC counts increased, decreased, or remained unchanged between baseline and PD in 4, 6, and 16 out of 26 patients, respectively. Overall, there was no significant change in CTC counts from baseline to C1D8 (mean rank: 8.29 versus 4; *p* = 0.133, Wilcoxon *t*-test), or to PD (mean rank: 6.5 vs. 4; *p* = 0.239).

CTC clusters (defined as clusters of 2 or more CTCs) were also identified in 3/42 patients (7.1%) only at baseline; single CTCs were in parallel detected in all these patients. The composition of CTC clusters was as follows: #patient 1: one cluster of 6 cells, #patient 2: one cluster of 10 cells, #patient 3: one cluster of 4 cells & three clusters of 2 cells. The inclusion of CTC clusters in the analysis did not alter the status of these patients regarding positivity for CTCs or particular CTC phenotypes; therefore, hereafter, single CTCs and CTC clusters are referred to as one.

### 3.3. Phenotypic Analysis of CTCs during Eribulin Treatment and on Disease Progression

**Figure 1:**
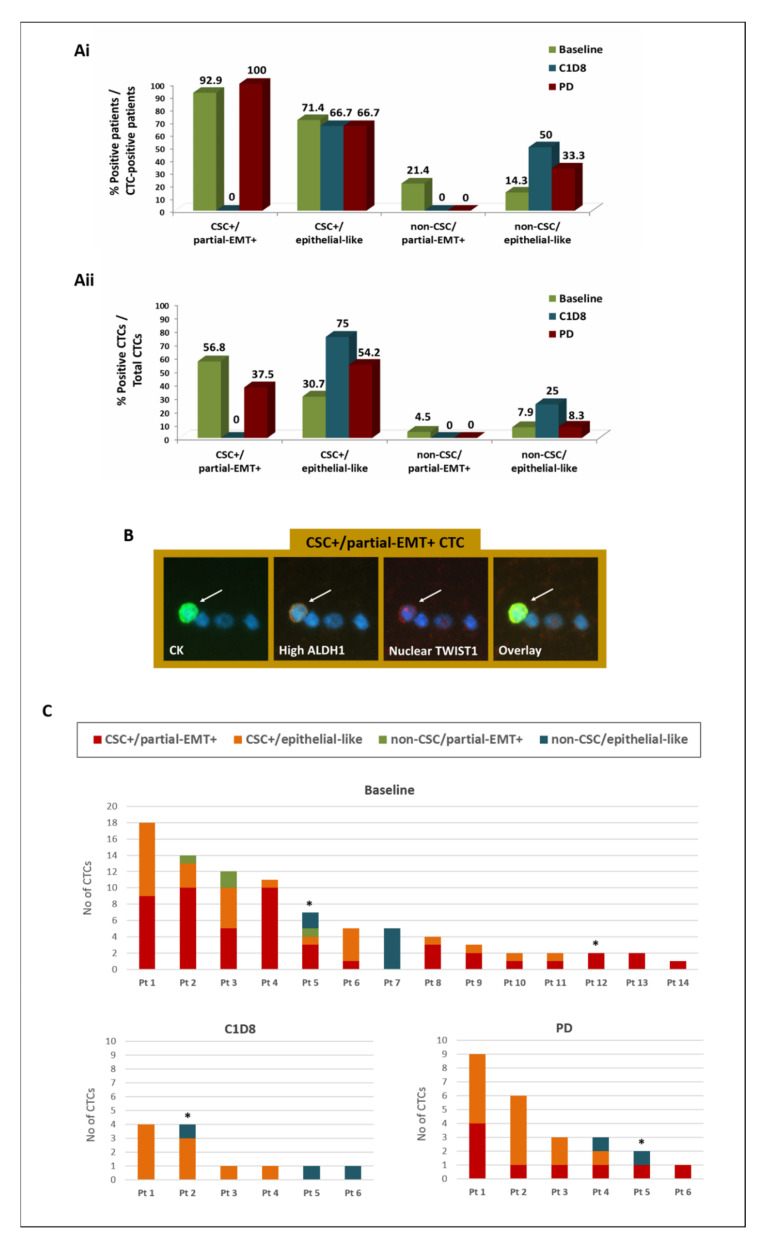
Distribution of the CSC-like and EMT-like phenotypes on CTCs of BC patients during eribulin treatment and on disease progression. (**Ai**) Percentage of patients harboring distinct CTC subsets; (**Aii**) Percentage of distinct CTC subsets per total CTCs; (**Β**) Representative image of a CSC+/partial-EMT+ CTC (CK-positive cell—arrow) among PBMCs (CK-negative cells) detected in the peripheral blood of a BC patient: CKs (green), ALDH1 (orange), TWIST1 (red) and DAPI (blue), Ariol microscopy system—200×; (**C**) Distribution of the number of distinct CTC subsets in each CTC-positive patient (patients treated with a reduced drug dosage are indicated with *). Abbreviations: CSC: cancer stem cell; EMT: epithelial to mesenchymal transition; C1D8: day 8 of 1st cycle of treatment; PD: disease progression.

CTC analysis at baseline of eribulin treatment revealed four distinct phenotypes: (a) CSC+/partial-EMT+, (b) CSC+/epithelial-like, (c) non-CSC/partial-EMT+, and (d) non-CSC/epithelial-like (Figure 1). CTCs expressing the CSC+/partial-EMT+ phenotype emerged as the most frequent subset at baseline, identified in 92.9% of CTC-positive patients and representing 56.8% of total CTCs (Figure 1(Ai,Aii)). A representative image of a CSC+/partial-EMT+ CTC identified in the PB of a BC patient is depicted in Figure 1B. The distribution of distinct CTC subsets identified in individual patients at different time points is shown in Figure 1C.

On C1D8, CSC+/partial-EMT+ CTCs and non-CSC/partial-EMT+ CTCs were completely absent, whereas the CSC+/epithelial-like and the non-CSC/epithelial-like phenotypes were enriched at the patient and/or CTC level (Figure 1A,C).

During disease progression (PD), CSC+/partial-EMT+ CTCs were detected again in all patients and represented 37.5% of CTCs (Figure 1A,C). In contrast, the non-CSC/partial-EMT+ phenotype remained undetectable. The CSC+/epithelial-like population was frequently detected on PD, representing 54.2% of CTCs (Figure 1A,C).

Individual analysis of the EMT and CSC phenotypes revealed that partial-EMT+ CTCs represented 61.3%, 0%, and 37.5% of total CTCs at baseline, C1D8 and PD, respectively, and their numbers significantly decreased from baseline to C1D8 (mean rank: 4.5 versus 0; *p* = 0.011, Wilcoxon *t*-test). On the other hand, the CSC-like phenotype was consistently expressed by 87.5%, 75%, and 91.7% of total CTCs at baseline, C1D8, and PD, respectively, with an insignificant reduction being recorded from baseline to C1D8 (mean rank: 7.6 versus 3.3; *p* = 0.074, Wilcoxon *t*-test).

The eribulin dosage administered to patients was further assessed to investigate whether the detection of CTCs and the persistence of the CSC-like phenotype following eribulin treatment could be the consequence of the administration of reduced drug doses. As described in Section 2, 34 patients received the full drug dosage (1.23 mg/m^2^), whereas eight patients received the reduced dose (0.97 mg/m^2^). Among the 14 patients with detectable CTCs at the baseline of treatment, only two patients were treated with the reduced dosage (Figure 1C; Pt5 and P12, marked with *); both of these patients harbored CSC-like CTCs and/or non-CSC-like CTCs at baseline; however, they had no CTCs detectable at C1D8 and on PD. In addition, among the 6 CTC-positive patients at C1D8 and on PD, 5 of them received the full drug dosage, whereas 1 patient only received the reduced dose from the start of therapy; this patient harbored CSC-like and non-CSC-like CTCs at both time points (Figure 1C; C1D8: Pt2; PD: Pt5, marked with *); however, no CTCs were detected in this patient at the start of treatment. These results indicate that the administration of reduced drug doses is not correlated with the detection of CTCs or CSC-like CTCs following eribulin treatment.

### 3.4. Clinical Relevance of the Detection and the CSC/Partial-EMT Phenotype of CTCs in BC Patients Treated with Eribulin Mesylate

#### 3.4.1. Correlation with Patient and Disease Characteristics

CTC detection at baseline was more frequently encountered among younger patients (below average, 60 years; 55% versus 13.6% of patients, *p* = 0.008), triple-negative subtype (61.5% versus 20.7%; *p* = 0.009), and liver metastases (in 48% versus 11.8%; *p* = 0.014) (Fisher’s exact test). In addition, CTC detection at any time point was more frequent among patients experiencing PD as compared to those with partial response or stable disease at the end of treatment, albeit not reaching statistical significance (50% versus 18.2%; *p* = 0.137). No correlation was shown between the kinetics or phenotype of CTCs and clinicopathological characteristics or response to treatment.

#### 3.4.2. Correlation with Survival

**Figure 2:**
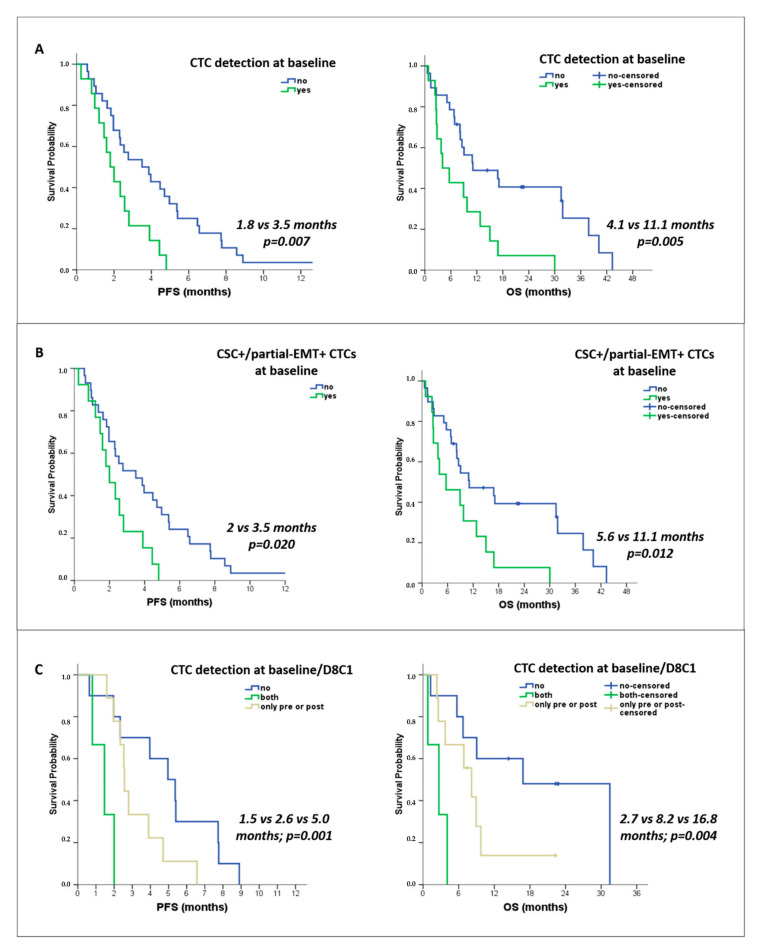
Correlation of the detection of CTCs and CSC/partial-EMT-like CTCs with patient outcomes. Kaplan–Meier survival curves for PFS and OS according to: (**A**) Baseline detection of CTCs (*n* = 42 patients), (**B**) Baseline detection of CSC+/partial-EMT+ CTCs (*n* = 42 patients), and (**C**) Combined CTC status at baseline and C1D8 (*n* = 22 patients).

Kaplan–Meier survival analysis revealed an association between CTC detection at baseline and shorter progression-free survival (median PFS: 1.8 versus 3.5 months; *p* = 0.007) and overall survival (median OS: 4.1 vs. 11.1 months; *p* = 0.005) (Figure 2A). Reduced survival rates were also recorded for patients with detectable CSC+/partial-EMT+ CTCs at baseline (median PFS: 2 vs. 3.5 months; *p* = 0.020; median OS: 5.6 vs. 11.1 months; *p* = 0.012) (Figure 2B).

Subgroup analysis of patients with available samples at baseline and C1D8 revealed that patients with detectable CTCs at both time points had lower survival rates as compared to those harboring CTCs at a one-time point only and those remaining CTC-negative (median PFS: 1.5 versus 2.6 vs. 5 months, *p* = 0.001; median OS: 2.7 vs. 8.2 vs. 16.8 months, *p* = 0.004) (Figure 2C). In addition, a shorter PFS was observed among patients with detectable CTCs (median: 2.3 vs. 3.9 months, respectively, *p* = 0.024), and specifically CSC+/partial-EMT+ CTCs (median: 2.3 vs. 3.5 months; *p* = 0.026), at any of the three time points (baseline, C1D8, PD).

#### 3.4.3. Assessment of the Risk of Disease Progression and Death

Univariate cox-regression analysis revealed that patients with detectable CTCs at baseline were at high risk for disease progression (HR: 2.565, *p* = 0.010) and death (HR: 2.727, *p* = 0.007) (Table 3). The detection of CSC+/partial-EMT+ CTCs was also associated with high risk of disease progression (HR: 2.297, *p* = 0.024] and death (HR: 2.467, *p* = 0.016) (Table 3).

In multivariate cox-regression analysis, the triple-negative subtype (HR: 2.538, *p* = 0.032) and brain metastases (HR: 2.868, *p* = 0.026) independently predicted the increased risk of disease progression. In addition, brain metastases (HR: 11.100 *p* = 0.000), skin metastases (HR: 8.331, *p* = 0.000), and the baseline detection of all CTCs (HR: 3.779, *p* = 0.001) or CSC+/partial-EMT+ CTCs (HR: 3.714, *p* = 0.001) emerged as independent risk factors for death (Table 3).

Subgroup analysis of patients with available samples at baseline and C1D8 revealed that the detection of CTCs at baseline (HR: 4.460, *p* = 0.010) and especially the persistence of CTCs at both time points (HR: 9.545, *p* = 0.014) were the only factors predicting high risk for progression (Univariate analysis, Table 4). An increased risk for death was also recorded among CTC-positive patients at baseline (HR: 5.109, *p* = 0.003) and those found CTC-positive at both time points (HR: 7.579, *p* = 0.031) (Table 4).

In multivariate analysis, brain metastases (HR: 3.888, *p* = 0.042) and baseline CTC detection (5.222, *p* = 0.003) emerged as independent risk factors for death (Table 4).

## 4. Discussion

Eribulin mesylate exhibits significant effects on cancer cell migration and metastatic seeding and, interestingly, has been shown to reverse the EMT state of BC cells [15] and to target the CSC-like BC subsets in preclinical studies [16]. In the current study, we used the liquid biopsy tool to investigate the effects of eribulin on CTCs of patients with metastatic BC, to identify CTC subsets that prevail during disease progression, and to evaluate the prognostic value of CTCs for eribulin-treated BC patients. The current study reveals for the first time the dynamic alterations in the CSC and EMT status of CTCs early on during eribulin treatment and on disease progression and identifies therapy-resistant CTC subsets. Notably, the results highlight the role of CTC assessment in the prediction of the outcome of patients with metastatic BC treated with eribulin mesylate.

In the current study, we initially investigated the possible changes in the frequency and the phenotype of CTCs eight days after the first administration of eribulin treatment (C1D8). The results revealed a minor decrease in the detection frequency and the number of CTCs from baseline to C1D8, thus implying that the CTC burden was not severely affected early on during eribulin treatment. However, interestingly we observed a notable variation in the distribution of distinct CTC phenotypes between baseline and C1D8. Specifically, CSC+/partial-EMT+ CTCs, which represented the most prevalent subset at baseline, were completely absent on C1D8. In addition, non-CSC/partial-EMT+ CTCs were also absent, while in contrast, the frequency of CSC+/epithelial-like CTCs and non-CSC/epithelial-like CTCs was relatively increased. Taken together, our results show that all partial-EMT+ CTCs were eradicated on C1D8, thus suggesting a complete reversal of the EMT state of CTCs. This finding is in accordance with the previously reported reversal of EMT on BC cells in vitro within eight days of eribulin treatment [15] and with the phenotypic switch observed following eribulin treatment in BC tissues [26] and CTCs [27,28]. These results also corroborate our previous findings in BC, showing a reduction of mesenchymal-like CTCs on C1D8 of eribulin treatment by the use of a different methodology [29].

More importantly, in the present study, we assessed for the first time the effect of eribulin on the CSC-like phenotype of CTCs, in view of the anti-CSC activity of eribulin that was recently reported in BC cell lines [16]. Our results demonstrate that despite the elimination of the CSC+/partial-EMT+ CTC subset on D8C1, the frequency of the CSC-like population of CTCs overall remained unchanged. This finding provides initial evidence that eribulin could not effectively target in vivo the CSC-like CTCs. We further confirmed that the persistence of CSC-like CTCs following treatment was not the consequence of the administration of reduced drug doses due to hematological and non-hematological toxicity. The discrepancy between our results and the in vitro evidence in Kurebayashi et al. [16] could be attributed to the relatively small number of CTCs analyzed, the significant heterogeneity of CTCs as compared to cell lines, and the different methodologies employed that could possibly identify different CSC-like populations displaying distinct properties. On the other hand, our findings corroborate preclinical findings in BC, showing that eribulin resistance is promoted by the activation of the PI3K/AKT pathway [21], which holds a key role in maintaining the stemness of BC cells [22], and that the combination of eribulin with PI3K inhibition has synergistic antitumor effects by reducing the breast CSC population [30]. The role of CSCs in primary and acquired resistance to eribulin needs further investigation in BC patients, and analyses at the CTC level could be very informative in this regard.

To investigate further the phenotypic profile of therapy-resistant CTC subsets, we also analyzed CTCs from the same patients using blood samples obtained on disease progression following eribulin treatment. The results clearly suggest that CTCs with CSC traits prevail on disease progression. More particularly, CSC+/epithelial-like CTCs were frequently detected, whereas CSC+/partial-EMT+ CTCs (which were previously eliminated on C1D8) were identified again in all patients during disease progression. Taken together, these results underscore a prominent role of CSC+ CTCs in the resistance to eribulin mesylate therapy. This could provide an explanation for the absence of significant improvement in PFS/OS of patients with eribulin-treated advanced BC [17,18] and could set the basis for future treatment strategies combining eribulin with anti-CSCs agents. Our results also suggest that even though all partial-EMT+ CTCs were eradicated early on during eribulin treatment, however, specifically, the CSC+/partial-EMT+ subset reappeared and prevailed during disease progression. A growing body of evidence supports that EMT and stemness represent two dynamic and reversible states of tumor cells which can frequently shift under therapy pressure to develop drug resistance ultimately [9,31]. We here hypothesize that the phenotypic switch of CTCs during eribulin treatment may be part of their mechanism to acquire resistance. Our previous studies in BC also highlight the CSC+/partial-EMT+ CTC subset as an aggressive and chemoresistant cell population that prevailed in patients with metastatic as compared to early-stage BC [11] and was further enriched at the end of first-line chemotherapy [14]. The results presented herein underline the unique opportunity provided by the longitudinal CTC analysis to identify the specific biological features of CTCs during the course of treatment and disease progression, thus expanding our knowledge of the mechanisms underlying therapy resistance and metastasis.

Importantly, the current study provides evidence for the prognostic value of CTCs in BC patients treated with eribulin treatment. Specifically, the detection of CTCs at baseline was associated with reduced PFS and OS rates and with a high risk for disease progression and independently predicted a high risk for death. Interestingly, CSC+/partial-EMT+ CTCs emerged as the only subset associated with poor patient outcomes. These results are an important contribution to the existing data showing the unfavorable prognostic role of CTCs and mesenchymal-like CTC subsets in the eribulin-treated metastatic BC setting [27,28]. In our previous report, the detection of mesenchymal-like CTC clusters was associated with a high risk of death in patients with metastatic BC treated with eribulin [29]. Instead, we here observed that the detection of clusters did not add any prognostic value to that obtained by the single CTC analysis, which might be explained by the low detection frequency of clusters and the different methodologies employed. The results presented herein provide a clear indication that CTCs, and particularly the CSC+/partial-EMT+ CTC subset, hold independent prognostic relevance for BC patients treated with eribulin mesylate. This finding is also in accordance with our previous report that features CSC+/partial-EMT+ CTCs as an independent adverse prognostic factor for patients receiving first-line chemotherapy, especially those with HER2-negative disease [14].

It is currently under investigation whether the sequential CTC enumeration during the course of treatment can advance the monitoring of treatment efficacy in patients with different malignancies [5,32,33,34]. Interestingly, here we did not identify any association between the dynamic changes of CTCs or distinct CTC subsets and response to eribulin treatment. In accordance, our previous and other studies also failed to show a clear correlation between the kinetics of CTCs or EMT-like CTCs and eribulin efficacy [27,29] while in contrast, Horimoto et al. reported a significant increase in CTC counts during treatment among patients experiencing disease progression [27]. Importantly, we herein show that the persistent detection of CTCs at baseline and on C1D8 was associated with shorter PFS and OS rates and a high risk for disease progression and death. It is worth mentioning that CTC monitoring improved the prognostic information obtained by the baseline CTC assessment with regards to predicting PFS and the risk for disease progression. In accordance with this finding, the longitudinal CTC enumeration improved the prognostication and monitoring of patients with metastatic BC starting first-line systemic therapy [5]. On the other hand, the CTC-based monitoring of treatment efficacy did not show any clinical utility for metastatic BC patients beyond the third line of chemotherapy [35]. However, regardless of the possibility of adding prognostic value, it is worth considering the significance of monitoring the phenotypic profile of CTCs to uncover mechanisms of resistance and disease progression.

As limitations of our study, it is important to mention that the evaluation of additional markers, other than ALDH1 and TWIST1, as well as functional experiments, would be required for a more accurate assessment of the CSC/EMT-like state of CTCs. In addition, even though the results provide important indications for phenotypic changes in CTCs during treatment, however, it was not possible to verify whether a specific phenotypic shift occurred on an individual CTC. Functional studies, including sorting of single CTCs, expansion in CTC cultures, and in vitro treatment with eribulin mesylate, would help to estimate a phenotypic transition at the single cell level. Moreover, the longitudinal CTC analysis was limited by the low availability of samples on C1D8 and PD, similar to the previous studies that included even smaller patient cohorts [27,28,29]. These limitations preclude any valid conclusions about the clinical relevance of specific phenotypic changes in CTCs. In addition, CD45 was not included in the immunofluorescence panel for CTC analysis due to the inability of our method to combine more than four markers (cytokeratins, ALDH1, TWIST1, DAPI); consequently, the identification of CTCs was based on the expression of cytokeratins and morphologic criteria as described in numerous reports from our group and others [23,24,36,37]. However, our previous studies in independent cohorts of patients with BC and healthy volunteers [11,13,38,39,40,41] revealed that there were no cytokeratin+/CD45+ cells evident in the peripheral blood of patients or healthy donors, thus indicating the high specificity of our method.

To summarize, the current study provides for the first time evidence that CTCs temporarily lose their EMT-like features early on during eribulin treatment; however, they retain their CSC traits. In addition, the CSC-like phenotype prevails during disease progression, thus indicating a prominent role of CSC-like CTCs in the resistance to eribulin mesylate. Special emphasis is given to the CSC+/partial-EMT+ CTC subset, which is initially eliminated during treatment and subsequently reappears during disease progression, and significantly correlates with poor patient outcomes. These findings expand our knowledge of the mechanisms underlying resistance to eribulin and provide the rationale for combining eribulin with anti-CSCs agents. The current study also provides clear indications that CTC detection and monitoring hold a significant prognostic role for BC patients receiving eribulin treatment. Future studies, including larger patient cohorts, would help to confirm the prognostic value of CTCs for eribulin-treated patients and to assess their role in monitoring treatment efficacy. Interestingly, the combination of eribulin with anti-PD-1 immunotherapy has shown promising antitumor activity in metastatic BC, especially in the triple-negative subtype [42,43,44]. Moreover, the role of eribulin treatment as monotherapy is currently under investigation in the neoadjuvant BC setting [45]. Reliable and readily accessible biomarkers would be of utmost importance to extend the use of eribulin mesylate in new therapeutic strategies for BC. Based on the current results, we consider that CTC-based analyses may largely contribute towards this direction.

## 5. Conclusions

The current study provides initial evidence for the involvement of CSC-like CTCs in resistance to eribulin and highlights the value of the longitudinal CTC phenotyping for the elucidation of the mechanisms underlying therapy resistance and metastasis. The results also underline the role of CTC detection and monitoring as a liquid biopsy tool for the prediction of the outcome of patients with metastatic BC treated with eribulin mesylate.

## Figures and Tables

**Table 1:** Patient and disease characteristics.

Patients (*n* = 42)	*n* (%)
Age (years), median (range)	61 (34–78)
**Performance Status (PS)**	
0–1	35 (83.3)
2	7 (16.7)
**Histology**	
Ductal	33 (78.6)
Lobular	5 (11.9)
Mixed	2 (4.8)
Other	2 (4.8)
**Stage at diagnosis**	
I–III	33 (78.6)
IV	9 (21.4)
**Subtype**	
HR+/HER2−	22 (52.4)
Triple-negative	13 (31)
HER2+	7 (16.7)
**Prior chemotherapy for metastatic disease ^a^**	
Taxane-based	18 (42.9)
Anthracycline-based	1 (2.4)
Taxane/Anthracycline -based	16 (38.1)
Other	4 (9.5)
None	3 (7.1)
**Organs affected**	
Bones	27 (64.3)
Liver	25 (59.5)
Lung	20 (47.6)
Brain	6 (14.3)
Lymph nodes	15 (35.7)
Skin	7 (16.7)
**Line of Eribulin treatment**	
1–2	13 (31)
>2	29 (69)
**Response to eribulin treatment at first evaluation**	
Partial response	4 (9.5)
Stable disease	11 (26.2)
Progressive disease	20 (47.6)
Non-evaluable	7 (16.7)
**Disease status at the end of treatment**	
Partial response	1 (2.4)
Stable disease	10 (23.8)
Progressive disease	24 (57.1)
Non-evaluable	7 (16.7)

^a^ Patients with HER2-positive disease also received trastuzumab. Abbreviations: HR, hormone receptor.

**Table 2:** Distribution of CTCs in patients with metastatic BC at different time points during eribulin treatment and on disease progression.

	CTC Assessment		
*Patients*	Baseline	C1D8	PD
CTC-positive/total patients	14/42	6/22	6/26
Patient positivity	33.3%	27.3%	23.1%
***CTC counts***			
Sum (range)	88 (1–18)	12 (1–4)	24 (1–9)
Mean no of CTCs	6.29	2.00	4.00

Abbreviations: C1D8: day 8 of 1st cycle; PD: disease progression.

**Table 3:** Cox regression analysis for PFS and OS prediction in the entire population of patients with metastatic BC (*n* = 42).

Progression-Free Survival (PFS).	Univariate Analysis		Multivariate Analysis	
***Covariates***	***HR (95% CI)***	***p Value***	***HR (95% CI)***	***p Value***
Age (below vs. above average, 60 years)	1.738 (0.906–3.331)	0.096		
Performance status (2 vs. 0–1)	2.080 (0.889–4.865)	0.091		
Stage at diagnosis (IV vs. I–III)	1.563 (0.716–3.411)	0.262		
*Tumor subtype*				
Hormone receptor+/HER2−	reference		reference	
HER2+	1.894 (0.778–4.614)	0.159	1.894 (0.778–4.614)	0.159
Triple-negative	2.538 (1.086–5.936)	0.032 *	2.538 (1.086–5.936)	0.032 *
*Metastatic sites (yes vs. no)*				
Brain	2.570 (1.032–6.399)	0.043 *	2.868 (1.132–7.264)	0.026 *
Liver	1.394 (0.741–2.626)	0.483		
Lung	1.008 (0.544–1.870)	0.651		
Bones	1.153 (0.589–2.257)	0.679		
Lymph nodes	0.786 (0.403–1.536)	0.482		
Skin	1.059 (0.463–2.422)	0.891		
Line of eribulin treatment (>2 vs. 1–2)	0.902 (0.465–1.748)	0.759		
*CTC assessment* ^a^				
All CTC detection at baseline (yes vs. no)	2.565 (1.254–5.248)	0.010 *	2.034 (0.927–4.461)	0.076
CSC+/partial-EMT+ CTCs at baseline (yes vs. no)	2.297 (1.118–4.722)	0.024 *	1.784 (0.812–3.921)	0.150
**Overall Survival (OS)**	**Univariate Analysis**		**Multivariate Analysis**	
***Covariates***	***HR (95% CI)***	***p value***	***HR (95% CI)***	***p value***
Age (below vs. above average, 60 years)	1.116 (0.567–2.198)	0.750		
Performance status (2 vs. 0–1)	2.540 (1.019–6.332)	0.045 *		
Stage at diagnosis (IV vs. I–III)	0.608 (0.233–1.587)	0.309		
*Tumor Subtype*				
Hormone receptor+/HER2−	reference			
HER2+	0.671 (0.223–2.020)	0.478		
Triple-negative	1.729 (0.831–3.598)	0.143		
*Metastatic sites (yes vs. no)*				
Brain	5.854 (2.096–16.354)	0.001 *	11.100 (3.550–34.711)	0.000 *
Liver	1.124 (0.543–2.327)	0.754		
Lung	0.909 (0.463–1.786)	0.783		
Bones	1.735 (0.836–3.603)	0.139		
Lymph nodes	0.752 (0.368–1.539)	0.436		
Skin	4.339 (1.716–10.969)	0.002 *	8.331 (2.888–24.030)	0.000 *
Line of eribulin treatment (>2 vs. 1–2)	4.116 (0.493–34.392)	0.191		
*CTC assessment* ^a^				
All CTC detection at baseline (yes vs. no)	2.727 (1.315–5.656)	0.007 *	3.779 (1.737–8.222)	0.001 *
CSC+/partial-EMT+ CTCs at baseline (yes vs. no)	2.467 (1.187–5.128)	0.016 *	3.714 (1.684–8.191)	0.001 *

* Statistical significance at the *p* < 0.05 level. ^a^ CTC-related parameters were separately tested in multivariate analysis, following the one in ten rule (see Section 2).

**Table 4:** Cox regression analysis for PFS and OS prediction in metastatic BC patients who were eligible for CTC monitoring (*n* = 22).

Progression-Free Survival (PFS)	Univariate Analysis			
*Covariates*	*HR (95% CI)*	*p Value*		
Age (below vs. above average, 60 years)	1.818 (0.735–4.496)	0.196		
Performance status (2 vs. 0–1)	1.819 (0.503–6.579)	0.362		
Stage at diagnosis (IV vs. I–III)	1.832 (0.623–5.391)	0.272		
*Tumor subtype*				
Hormone receptor+/HER2−	reference			
HER2+	1.142 (0.311–4.186)	0.842		
Triple-negative	2.613 (0.909–7.516)	0.075		
*Metastatic sites (yes vs. no)*				
Brain	2.418 (0.751–7.787)	0.139		
Liver	1.045 (0.430–2.539)	0.923		
Lung	1.064 (0.450–2.515)	0.888		
Bones	1.265 (0.502–3.189)	0.618		
Lymph nodes	0.793 (0.326–1.929)	0.609		
Skin	1.155 (0.413–3.235)	0.783		
Line of eribulin treatment (>2 vs. 1–2)	1.463 (0.487–4.399)	0.498		
*CTC assessment*				
CTC detection at baseline (yes vs. no)	4.460 (1.422–13.987)	0.010 *		
CTC detection at baseline and C1D8				
Negative	reference			
Positive in one time point	2.731 (0.995–7.498)	0.051		
Positive in both time points	9.545 (1.556–58.189)	0.014 *		
**Overall Survival (OS)**	**Univariate Analysis**		**Multivariate Analysis**	
***Covariates***	***HR (95% CI)***	***p value***	***HR (95% CI)***	***p value***
Age (below vs. above average, 60 years)	3.173 (1.045–9.637)	0.042 *		
Performance status (2 vs. 0–1)	1.038 (0.233–4.626)	0.961		
Stage at diagnosis (IV vs. I–III)	0.963 (0.268–3.465)	0.954		
*Tumor subtype*				
Hormone receptor+/HER2−	reference			
HER2+	0.345 (0.042–2.838)	0.163		
Triple-negative	1.729 (0.831–3.598)	0.092		
*Metastatic sites (yes vs. no)*				
Brain	3.787 (1.058–13.557)	0.041 *	3.888 (1.050–14.399)	0.042 *
Liver	2.074 (0.742–5.802)	0.164		
Lung	0.897 (0.318–2.530)	0.838		
Bones	3.237 (0.998–10.502)	0.050		
Lymph nodes	0.990 (0.357–2.748)	0.985		
Skin	2.893 (0.934–8.955)	0.065		
Line of eribulin treatment (>2 vs. 1–2)	1.023 (0.324–3.226)	0.969		
*CTC assessment* ^a^				
CTC detection at baseline (yes vs. no)	5.109 (1.716–15.210)	0.003 *	5.222 (1.723–15.828)	0.003 *
CTC detection at baseline and C1D8				
Negative	reference		reference	
Positive in one time point	2.805 (0.888–8.859)	0.079	2.675 (0.840–8.518)	0.096
Positive in both time points	7.579 (1.207–47.603)	0.031 *	4.355 (0.595–31.880)	0.147

* Statistical significance at the *p* < 0.05 level. ^a^ CTC-related parameters were separately tested in multivariate analysis.

## Data Availability

The data presented in this study are available on request from the corresponding author. The data are not publicly available due to privacy/ethical restrictions.

## References

[1] Sung H., Ferlay J., Siegel R.L., Laversanne M., Soerjomataram I., Jemal A., Bray F. (2021). Global Cancer Statistics 2020: GLOBOCAN Estimates of Incidence and Mortality Worldwide for 36 Cancers in 185 Countries. CA Cancer J. Clin..

[2] Dillekas H., Rogers M.S., Straume O. (2019). Are 90% of deaths from cancer caused by metastases?. Cancer Med..

[3] Castro-Giner F., Aceto N. (2020). Tracking cancer progression: From circulating tumor cells to metastasis. Genome Med..

[4] Cristofanilli M., Pierga J.Y., Reuben J., Rademaker A., Davis A.A., Peeters D.J., Fehm T., Nole F., Gisbert-Criado R., Mavroudis D. (2019). The clinical use of circulating tumor cells (CTCs) enumeration for staging of metastatic breast cancer (MBC): International expert consensus paper. Crit. Rev. Oncol. Hematol..

[5] Larsson A.M., Jansson S., Bendahl P.O., Levin Tykjaer Jorgensen C., Loman N., Graffman C., Lundgren L., Aaltonen K., Ryden L. (2018). Longitudinal enumeration and cluster evaluation of circulating tumor cells improve prognostication for patients with newly diagnosed metastatic breast cancer in a prospective observational trial. Breast Cancer Res..

[6] Bidard F.C., Jacot W., Kiavue N., Dureau S., Kadi A., Brain E., Bachelot T., Bourgeois H., Goncalves A., Ladoire S. (2021). Efficacy of Circulating Tumor Cell Count-Driven vs Clinician-Driven First-line Therapy Choice in Hormone Receptor-Positive, ERBB2-Negative Metastatic Breast Cancer: The STIC CTC Randomized Clinical Trial. JAMA Oncol..

[7] Baccelli I., Schneeweiss A., Riethdorf S., Stenzinger A., Schillert A., Vogel V., Klein C., Saini M., Bauerle T., Wallwiener M. (2013). Identification of a population of blood circulating tumor cells from breast cancer patients that initiates metastasis in a xenograft assay. Nat. Biotechnol..

[8] Shibue T., Weinberg R.A. (2017). EMT, CSCs, and drug resistance: The mechanistic link and clinical implications. Nat. Rev. Clin. Oncol..

[9] Yu M., Bardia A., Wittner B.S., Stott S.L., Smas M.E., Ting D.T., Isakoff S.J., Ciciliano J.C., Wells M.N., Shah A.M. (2013). Circulating breast tumor cells exhibit dynamic changes in epithelial and mesenchymal composition. Science.

[10] Theodoropoulos P.A., Polioudaki H., Agelaki S., Kallergi G., Saridaki Z., Mavroudis D., Georgoulias V. (2010). Circulating tumor cells with a putative stem cell phenotype in peripheral blood of patients with breast cancer. Cancer Lett..

[11] Papadaki M.A., Kallergi G., Zafeiriou Z., Manouras L., Theodoropoulos P.A., Mavroudis D., Georgoulias V., Agelaki S. (2014). Co-expression of putative stemness and epithelial-to-mesenchymal transition markers on single circulating tumour cells from patients with early and metastatic breast cancer. BMC Cancer.

[12] Papadaki M.A., Aggouraki D., Vetsika E.K., Xenidis N., Kallergi G., Kotsakis A., Georgoulias V. (2021). Epithelial-to-mesenchymal Transition Heterogeneity of Circulating Tumor Cells and Their Correlation with MDSCs and Tregs in HER2-negative Metastatic Breast Cancer Patients. Anticancer Res..

[13] Kallergi G., Papadaki M.A., Politaki E., Mavroudis D., Georgoulias V., Agelaki S. (2011). Epithelial to mesenchymal transition markers expressed in circulating tumour cells of early and metastatic breast cancer patients. Breast Cancer Res..

[14] Papadaki M.A., Stoupis G., Theodoropoulos P.A., Mavroudis D., Georgoulias V., Agelaki S. (2019). Circulating Tumor Cells with Stemness and Epithelial-to-Mesenchymal Transition Features Are Chemoresistant and Predictive of Poor Outcome in Metastatic Breast Cancer. Mol. Cancer Ther..

[15] Yoshida T., Ozawa Y., Kimura T., Sato Y., Kuznetsov G., Xu S., Uesugi M., Agoulnik S., Taylor N., Funahashi Y. (2014). Eribulin mesilate suppresses experimental metastasis of breast cancer cells by reversing phenotype from epithelial-mesenchymal transition (EMT) to mesenchymal-epithelial transition (MET) states. Br. J. Cancer.

[16] Kurebayashi J., Kanomata N., Yamashita T., Shimo T., Moriya T. (2016). Antitumor and anticancer stem cell activities of eribulin mesylate and antiestrogens in breast cancer cells. Breast Cancer.

[17] Cortes J., O’Shaughnessy J., Loesch D., Blum J.L., Vahdat L.T., Petrakova K., Chollet P., Manikas A., Dieras V., Delozier T. (2011). Eribulin monotherapy versus treatment of physician’s choice in patients with metastatic breast cancer (EMBRACE): A phase 3 open-label randomised study. Lancet.

[18] Kaufman P.A., Awada A., Twelves C., Yelle L., Perez E.A., Velikova G., Olivo M.S., He Y., Dutcus C.E., Cortes J. (2015). Phase III open-label randomized study of eribulin mesylate versus capecitabine in patients with locally advanced or metastatic breast cancer previously treated with an anthracycline and a taxane. J. Clin. Oncol..

[19] Twelves C., Cortes J., Vahdat L., Olivo M., He Y., Kaufman P.A., Awada A. (2014). Efficacy of eribulin in women with metastatic breast cancer: A pooled analysis of two phase 3 studies. Breast Cancer Res. Treat..

[20] Aogi K., Watanabe K., Kitada M., Sangai T., Ohtani S., Aruga T., Kawagichi H., Fujisawa T., Maeda S., Morimoto T. (2021). Clinical usefulness of eribulin as first- or second-line chemotherapy for recurrent HER2-negative breast cancer: A randomized phase II study (JBCRG-19). Int. J. Clin. Oncol..

[21] Gris-Oliver A., Ibrahim Y.H., Rivas M.A., Garcia-Garcia C., Sanchez-Guixe M., Ruiz-Pace F., Viaplana C., Perez-Garcia J.M., Llombart-Cussac A., Grueso J. (2021). PI3K activation promotes resistance to eribulin in HER2-negative breast cancer. Br. J. Cancer.

[22] Teng X., Hayashida T., Murata T., Nagayama A., Seki T., Takahashi M., Kitagawa Y. (2021). A transposon screen identifies enhancement of NF-kappaB pathway as a mechanism of resistance to eribulin. Breast Cancer.

[23] Papadaki M.A., Koutsopoulos A.V., Tsoulfas P.G., Lagoudaki E., Aggouraki D., Monastirioti A., Koutoulaki C., Apostolopoulou C.A., Merodoulaki A.C., Papadaki C. (2020). Clinical Relevance of Immune Checkpoints on Circulating Tumor Cells in Breast Cancer. Cancers.

[24] Papadaki M.A., Monastirioti A., Apostolopoulou C.A., Aggouraki D., Papadaki C., Michaelidou K., Vassilakopoulou M., Alexakou K., Mavroudis D., Agelaki S. (2022). TLR4 and pSTAT3 Expression on Circulating Tumor Cells (CTCs) and Immune Cells in the Peripheral Blood of Breast Cancer Patients: Prognostic Implications. Cancers.

[25] Papadaki M.A., Sotiriou A.I., Vasilopoulou C., Filika M., Aggouraki D., Tsoulfas P.G., Apostolopoulou C.A., Rounis K., Mavroudis D., Agelaki S. (2020). Optimization of the Enrichment of Circulating Tumor Cells for Downstream Phenotypic Analysis in Patients with Non-Small Cell Lung Cancer Treated with Anti-PD-1 Immunotherapy. Cancers.

[26] Kashiwagi S., Asano Y., Goto W., Takada K., Takahashi K., Hatano T., Tanaka S., Takashima T., Tomita S., Motomura H. (2018). Mesenchymal-epithelial Transition and Tumor Vascular Remodeling in Eribulin Chemotherapy for Breast Cancer. Anticancer Res..

[27] Horimoto Y., Tokuda E., Murakami F., Uomori T., Himuro T., Nakai K., Orihata G., Iijima K., Togo S., Shimizu H. (2018). Analysis of circulating tumour cell and the epithelial mesenchymal transition (EMT) status during eribulin-based treatment in 22 patients with metastatic breast cancer: A pilot study. J. Transl. Med..

[28] Ito M., Horimoto Y., Tokuda E., Murakami F., Uomori T., Himuro T., Nakai K., Orihata G., Iijima K., Saito M. (2019). Impact of circulating tumour cells on survival of eribulin-treated patients with metastatic breast cancer. Med. Oncol..

[29] Polioudaki H., Mala A., Gkimprixi E., Papadaki M.A., Chantziou A., Tzardi M., Mavroudis D., Agelaki S., Theodoropoulos P.A. (2020). Epithelial/Mesenchymal Characteristics and PD-L1 Co-Expression in CTCs of Metastatic Breast Cancer Patients Treated with Eribulin: Correlation with Clinical Outcome. Cancers.

[30] Rajput S., Guo Z., Li S., Ma C.X. (2019). PI3K inhibition enhances the anti-tumor effect of eribulin in triple negative breast cancer. Oncotarget.

[31] Polyak K., Weinberg R.A. (2009). Transitions between epithelial and mesenchymal states: Acquisition of malignant and stem cell traits. Nat. Rev. Cancer.

[32] Papadaki M.A., Messaritakis I., Fiste O., Souglakos J., Politaki E., Kotsakis A., Georgoulias V., Mavroudis D., Agelaki S. (2021). Assessment of the Efficacy and Clinical Utility of Different Circulating Tumor Cell (CTC) Detection Assays in Patients with Chemotherapy-Naive Advanced or Metastatic Non-Small Cell Lung Cancer (NSCLC). Int. J. Mol. Sci..

[33] Shishido S.N., Carlsson A., Nieva J., Bethel K., Hicks J.B., Bazhenova L., Kuhn P. (2019). Circulating tumor cells as a response monitor in stage IV non-small cell lung cancer. J. Transl. Med..

[34] Kiniwa Y., Nakamura K., Mikoshiba A., Ashida A., Akiyama Y., Morimoto A., Okuyama R. (2021). Usefulness of monitoring circulating tumor cells as a therapeutic biomarker in melanoma with BRAF mutation. BMC Cancer.

[35] Cabel L., Berger F., Cottu P., Loirat D., Rampanou A., Brain E., Cyrille S., Bourgeois H., Kiavue N., Deluche E. (2021). Clinical utility of circulating tumour cell-based monitoring of late-line chemotherapy for metastatic breast cancer: The randomised CirCe01 trial. Br. J. Cancer.

[36] Agelaki S., Kalykaki A., Markomanolaki H., Papadaki M.A., Kallergi G., Hatzidaki D., Kalbakis K., Mavroudis D., Georgoulias V. (2015). Efficacy of Lapatinib in Therapy-Resistant HER2-Positive Circulating Tumor Cells in Metastatic Breast Cancer. PLoS ONE.

[37] Meng S., Tripathy D., Frenkel E.P., Shete S., Naftalis E.Z., Huth J.F., Beitsch P.D., Leitch M., Hoover S., Euhus D. (2004). Circulating tumor cells in patients with breast cancer dormancy. Clin. Cancer Res..

[38] Kallergi G., Mavroudis D., Georgoulias V., Stournaras C. (2007). Phosphorylation of FAK, PI-3K, and impaired actin organization in CK-positive micrometastatic breast cancer cells. Mol. Med..

[39] Spiliotaki M., Mavroudis D., Kokotsaki M., Vetsika E.K., Stoupis I., Matikas A., Kallergi G., Georgoulias V., Agelaki S. (2018). Expression of insulin-like growth factor-1 receptor in circulating tumor cells of patients with breast cancer is associated with patient outcomes. Mol. Oncol..

[40] Kallergi G., Konstantinidis G., Markomanolaki H., Papadaki M.A., Mavroudis D., Stournaras C., Georgoulias V., Agelaki S. (2013). Apoptotic circulating tumor cells in early and metastatic breast cancer patients. Mol. Cancer Ther..

[41] Kallergi G., Markomanolaki H., Giannoukaraki V., Papadaki M.A., Strati A., Lianidou E.S., Georgoulias V., Mavroudis D., Agelaki S. (2009). Hypoxia-inducible factor-1alpha and vascular endothelial growth factor expression in circulating tumor cells of breast cancer patients. Breast Cancer Res..

[42] Tolaney S.M., Barroso-Sousa R., Keenan T., Li T., Trippa L., Vaz-Luis I., Wulf G., Spring L., Sinclair N.F., Andrews C. (2020). Effect of Eribulin With or Without Pembrolizumab on Progression-Free Survival for Patients With Hormone Receptor-Positive, ERBB2-Negative Metastatic Breast Cancer: A Randomized Clinical Trial. JAMA Oncol..

[43] Keenan T.E., Guerriero J.L., Barroso-Sousa R., Li T., O’Meara T., Giobbie-Hurder A., Tayob N., Hu J., Severgnini M., Agudo J. (2021). Molecular correlates of response to eribulin and pembrolizumab in hormone receptor-positive metastatic breast cancer. Nat. Commun..

[44] Tolaney S.M., Kalinsky K., Kaklamani V.G., D’Adamo D.R., Aktan G., Tsai M.L., O’Regan R.M., Kaufman P.A., Wilks S.T., Andreopoulou E. (2021). Eribulin Plus Pembrolizumab in Patients with Metastatic Triple-Negative Breast Cancer (ENHANCE 1): A Phase Ib/II Study. Clin. Cancer Res..

[45] Pascual T., Oliveira M., Villagrasa P., Ortega V., Pare L., Bermejo B., Morales S., Amillano K., Lopez R., Galvan P. (2021). Neoadjuvant eribulin in HER2-negative early-stage breast cancer (SOLTI-1007-NeoEribulin): A multicenter, two-cohort, non-randomized phase II trial. NPJ Breast Cancer.

